# Glucose-only Therapy for Potassium Reduction: A Scoping Review

**DOI:** 10.1016/j.xkme.2025.101161

**Published:** 2025-10-29

**Authors:** Samuel Ford, Ian Coombes, Julian Williams, Claire Bertenshaw, Adam La Caze

**Affiliations:** 1 Department of Health and Behavioural Sciences, The University of Queensland, Brisbane, Australia; 2 Department of Medicine, The University of Queensland, Brisbane, Australia; 3 Pharmacy Department, Royal Brisbane and Women's Hospital, Brisbane, Australia; 4 Emergency and Trauma Centre, Royal Brisbane and Women's Hospital, Brisbane, Australia

**Keywords:** Diabetes mellitus, electrolyte management, endogenous insulin, glucose-only therapy, hyperkalemia, hypoglycemia, insulin, potassium, scoping review, serum potassium

## Abstract

Insulin-dextrose therapy is widely used to manage hyperkalemia but carries a significant risk of iatrogenic hypoglycemia. Glucose alone may reduce serum potassium by stimulating endogenous insulin release, potentially offering an insulin sparing alternative. This scoping review examined studies evaluating the effect of oral or intravenous glucose, without concurrent insulin, on serum or plasma potassium in adults. Nonhuman studies, nonpeer-reviewed sources, and studies where diabetes status could not be determined were excluded. Of 1,105 records screened, 30 met inclusion criteria. Most studies were small and conducted before 2,000, involving healthy individuals and patients with various conditions. A narrative synthesis was performed, with findings categorized by diabetes status and intervention type. Potassium responses varied: in nondiabetic individuals, glucose-only therapy generally lowered potassium, often to a clinically meaningful extent. In contrast, potassium often rose in insulin-dependent individuals. Methodological limitations were common, including small sample sizes, inconsistent statistical reporting, lack of control groups, and limited inclusion of hyperkalemic patients. Although glucose-only therapy may be effective in nondiabetic populations, evidence in people with diabetes is inconsistent and the evidence base is limited. Further rigorous, controlled studies are needed to clarify the role of glucose-only therapy in hyperkalemia management and its impact on hypoglycemia risk.

Plain-language Summary

Hyperkalemia, a condition where there is too much potassium in the blood, is often treated with insulin and glucose. But insulin can cause low blood sugar (hypoglycemia), which can be harmful. This review examined whether using glucose alone—without insulin—could lower potassium safely. We found that in people without diabetes, glucose-only therapy often lowered potassium. But in people with diabetes, the results were mixed and, in some cases, potassium levels increased. Most studies included in this review were small and had design limitations, highlighting the need for further research.

Acute hyperkalemia is a common and potentially life-threatening electrolyte abnormality associated with cardiac arrhythmias and cardiac arrest.^1^ Potassium homeostasis is tightly regulated by a combination of hormonal, renal, and cellular mechanisms to maintain serum potassium within a narrow range.^2^^,^^3^ Insulin plays a critical role by promoting the intracellular shift of potassium through activation of Na^+^-K^+^-ATPases, particularly in skeletal muscle. Aldosterone and catecholamines further support this process through stimulation of cellular potassium uptake via distinct, complementary mechanisms.^4^ Aldosterone also plays a key role in promoting renal potassium excretion by enhancing secretion in the distal nephron. In addition to hormonal control, potassium balance is influenced by osmolarity and acid–base status. Hyperosmolar states, such as hyperglycemia, promote potassium efflux via solvent drag and cell shrinkage. Mineral acidosis also drives potassium efflux, whereas organic acidosis has little effect.^5^

Insulin-dextrose therapy (IDT) is commonly used in the treatment of hyperkalemia to temporarily push potassium intracellularly until elimination strategies such as hemodialysis can be implemented. Despite co-administration of dextrose, hypoglycemia is commonly encountered. Recent estimates suggest the overall incidence of hypoglycemia following IDT to be 17.2%, with severe hypoglycemia estimated to be 5.4%.^6^ Lower pretreatment blood glucose is the strongest predictor of hypoglycemia following IDT; nondiabetic status also increases risk, likely due to lower baseline glucose.^7^

The use of glucose without insulin has long been postulated as an option to lower serum potassium in patients without diabetes mellitus.^8^ More recently, this approach has garnered renewed interest for its potential to reduce or eliminate the risk of iatrogenic hypoglycemia.^9^^,^^10^ The proposed mechanism is that endogenous insulin production alone will lower serum potassium by upregulation of the Na-K-ATPase pump in skeletal muscle. This mechanism is closely linked but independent of insulin-mediated glucose uptake and is functionally preserved in metabolic syndrome and type 2 diabetes.^3^^,^^11^ Given this independent mechanism, glucose-only therapy may be an effective treatment for hyperkalemia in patients with type 2 diabetes who have preserved β-cell function as well as people without diabetes. Tee et al^10^ have advocated for a glucose-only arm to be included in future clinical trials for stable, nondiabetic patients with moderate hyperkalemia.

Despite growing interest, the role of glucose-only therapy in hyperkalemia management remains debated. The UK Kidney Association currently recommends against its use, citing concerns regarding both safety and efficacy.^12^ Specifically, that hypertonic glucose may theoretically worsen hyperkalemia because of osmotic solute drag and that endogenous insulin levels may be insufficient to achieve an adequate potassium shift.

To our knowledge, no published review has systematically evaluated the effects of glucose-only administration on potassium blood levels. For these reasons, a scoping review was conducted to map the existing literature, assess the consistency of findings, and identify existing knowledge gaps.

## Methods

### Protocol and Registration

The protocol for this scoping review was registered with the Open Science Framework on August 20, 2024, and was developed in accordance with the Preferred Reporting Items for Systematic Reviews and Meta-Analyses extension for Scoping Reviews (PRISMA-ScR) guidelines (Table S1). The protocol is publicly available at https://osf.io/9vkpq.

### Research Questions

The primary research question guiding this scoping review is:

• Does the administration of oral or intravenous glucose, in the absence of insulin, lower serum/plasma potassium in adults?

Secondary research questions include:

• Does oral or intravenous glucose administration reliably and sufficiently promote endogenous insulin production?
• Does intravenous glucose administration increase plasma osmolarity?
• Have any studies found an increase in serum or plasma potassium following glucose administration?
• Have other safety endpoints (eg, extravasation, phlebitis, and fluid overload) been described following intravenous glucose administration?

### Search Strategy and Information Sources

A comprehensive search strategy was developed in collaboration with a research librarian and implemented across 4 major databases: PubMed (PubMed), Embase (Elsevier), Web of Science (Clarivate), and Cochrane Library & CENTRAL (Wiley). The search was designed to identify studies that examined the change in serum or plasma potassium following the administration of intravenous or oral glucose without insulin in human participants. Key articles were used to validate the search strategy. The full search strategy for each database is provided in Table S2 and was initially implemented on June 18, 2024. A repeat search on July 18, 2025 identified no additional eligible studies.

### Eligibility Criteria

Eligible studies measured a change in serum or plasma potassium in human participants following the administration of intravenous or oral glucose without insulin. All study types were considered, including randomized controlled trials, cohort studies, case series, and case reports, although short communications were not included. Studies were excluded if they were non-English, sourced from non-peer-reviewed literature, or conducted in nonhuman subjects. In addition, studies were excluded if diabetes status could not be determined.

### Study Selection

After database searches, results were exported to EndNote, and duplicates were removed. Citations were then imported into Covidence,^13^ where 2 independent reviewers performed an initial screening of titles and abstracts. Full texts of potentially eligible studies were reviewed by one reviewer, with consultation as needed. Discrepancies in screening were resolved through discussion or by a third reviewer if consensus could not be reached.

### Data Extraction

Data extraction was conducted using a tool in Microsoft Excel, which was validated on a subset of included studies. Extracted data included study design, setting, sample size, population demographics, intervention details, maximal change in potassium, and maximal insulin levels. Insulin concentrations were reported as mU/L, converted from μU/mL and pmol/L where necessary, and glucose concentrations were reported as mmol/L, converted from mg/dL.

Insulin levels reported in the included studies were derived from serum, plasma, and immunoreactive insulin measurements. Differences in assay methodology may influence the interpretation of insulin concentrations and this variability should be considered when comparing peak insulin levels across studies.

Where raw data were not reported in-text, Plot Digitizer software was used to extract numerical data from figures.^14^^,^^15^ One reviewer extracted all data, with a second reviewer independently verifying 20% for quality assurance. Divergences were discussed and resolved by consensus.

During data extraction, the terms insulin-dependent and noninsulin-dependent diabetes were used in place of type 1 and type 2 diabetes, as they reflect the terminology in older studies and more clearly indicate the presence or absence of endogenous insulin production relevant to glucose-only therapy.

When discussing specific studies, serum or plasma potassium is specified based on the terminology used in the study. However, in general discussions, the term potassium is used to refer to either sample type. Serum and plasma are not clinically interchangeable measurements as serum excludes clotting factors, whereas plasma includes them which may affect potassium concentrations.^16^ Nonetheless, the qualitative direction of change (eg, decrease) remains consistent across both, provided the same sample type is used for all measurements.

### Synthesis of Results

A narrative synthesis was conducted to summarize the effect of glucose on serum or plasma potassium levels. Thematic organization was used to structure the findings, grouped according to the research questions, patient demographics, intervention types, and safety endpoints. A formal meta-analysis was not undertaken because of marked heterogeneity in study designs, populations, interventions, and outcome measures, as well as concerns regarding methodological quality. To address these limitations, a risk-of-bias assessment was conducted using a simplified tool adapted from the JBI critical appraisal checklist for quasi-experimental studies. Methodological strengths and limitations are also discussed throughout.

## Results

**Figure 1:**
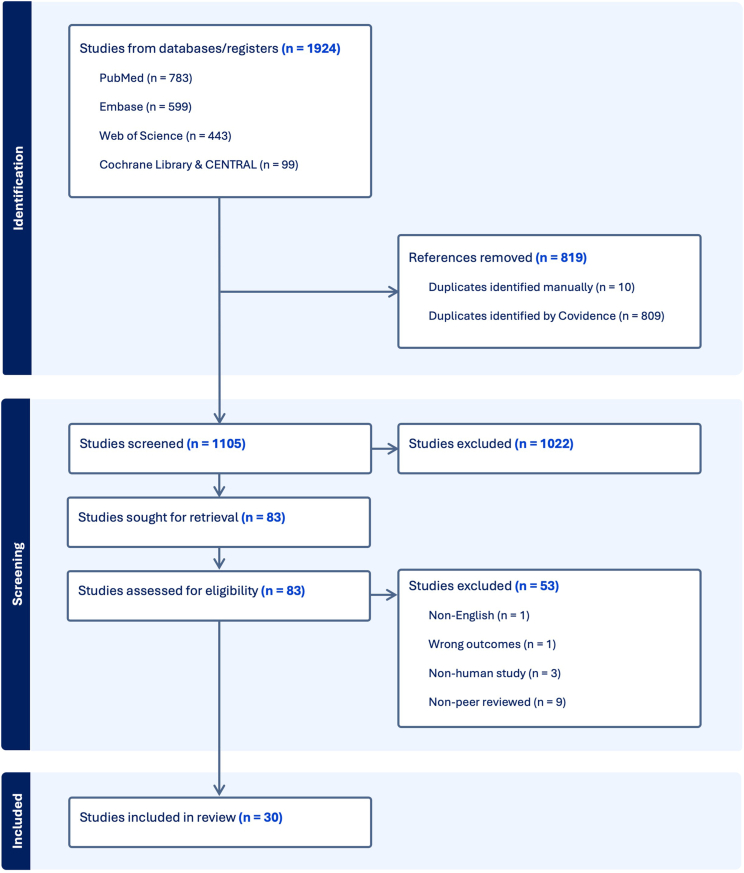
Preferred reporting items for systematic reviews and meta-analyses flowsheet of publications identified, included and excluded, and the reasons for exclusions.

The database search identified 1,924 articles, with 819 duplicates removed (Fig 1). After screening the titles and abstracts of 1,105 articles, 83 were selected for full-text review. Of these, 53 were excluded, resulting in a final inclusion of 30 studies.

Most studies were conducted before 2000 and had small sample sizes, ranging from 1 to 50 participants, with an average of ∼14. Table 1 summarizes some key characteristics of the included studies such as study design, country, year of publication, and sample size. Participants consisted of a mix of healthy individuals and those with clinical conditions such as diabetes mellitus, kidney disease, and pathologies related to the renin-angiotensin system. Both insulin-dependent and noninsulin-dependent participants were represented. Few studies included patients with hyperkalemia, and reporting of participants’ comorbidities and regular medications was frequently poor. Results from the risk-of-bias assessment are presented in Table S3.

**Table 1:** Summary of Characteristics of Included Studies

Items	Number of Studies
**Study design**	N = 30
Randomized controlled trial (RCT)	3
Quasi-experimental study (QES)	26
Case study (CS)	1
**Setting (country)**	N = 30
USA	12
Korea	1
South Africa	1
Japan	2
Ireland	1
Italy	1
Hungary	9
United Kingdom	2
Australia	1
**Year of publication**	N = 30
1969	1
1970-1979	8
1980-1989	13
1990-1999	4
2005	1
2010-2019	2
2021	1
**Sample size**	N = 30
Minimum	1
Maximum	50

Oral glucose was the most common intervention, with doses ranging from 37.5 g to 150 g. Studies examining intravenous glucose predominantly used bolus dosing of 0.5 to 1 g/kg. Although most studies evaluated glucose as a standalone intervention, 2 studies co-administered potassium. For participants with diabetes, insulin and oral hypoglycemic agents were typically withheld on the day of the study.

Most studies were quasi-experimental and recruited healthy participants or volunteers as a control group, offering a comparison to patient populations with underlying health conditions (eg, diabetes). However, nearly all studies lacked a control intervention, with only one study including IDT as a comparator. Potassium levels were monitored for periods ranging from 60 to 240 minutes post-administration, with most studies measuring serum potassium rather than plasma potassium.

Approximately half of the studies measured insulin levels, but only two assessed changes in osmolarity. Electrocardiograms (ECGs) were rarely performed or reported, with only one study examining post-glucose ECG changes in a hyperkalemic cohort. Safety outcomes, including extravasation and thrombophlebitis, were not reported in any of the included studies, and the reporting of statistical significance was inconsistent.

**Figure 2:**
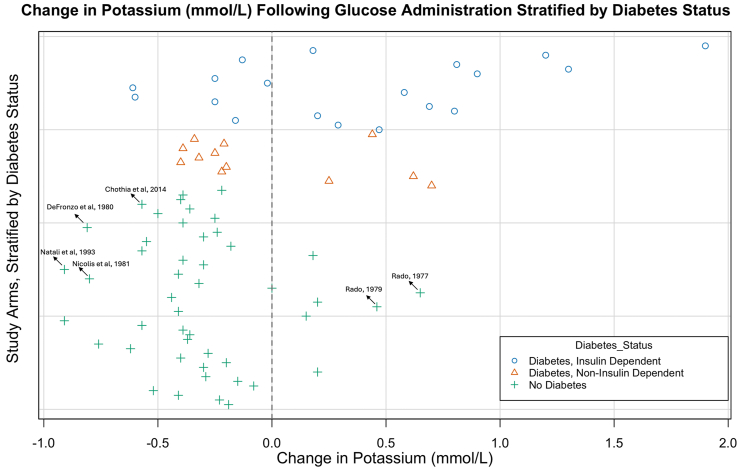
Data plot showing change in serum or plasma potassium concentration following oral or intravenous glucose administration, stratified by diabetes status. The x-axis shows the change in potassium (mmol/L) from baseline; the y-axis lists individual treatment arms (see Tables 1 & 2 for study details). Symbols denote diabetes status: ◯ insulin-dependent diabetes mellitus; △ noninsulin-dependent diabetes mellitus; and ✚ nondiabetic participants. The vertical dashed line at 0 mmol/L indicates no change from baseline. Several key studies discussed in the text are identified within the figure. This figure is designed to illustrate general trends only. The included studies are heterogeneous in design, intervention, and reporting quality, and most lack measures of variability. It should therefore be interpreted with caution.

Fig 2 provides an overview of the direction and magnitude of potassium change in each treatment arm, stratified by diabetes status. Given the heterogeneity and limited reporting quality of many included studies, this figure is intended to illustrate general trends only.

### Potassium Responses to Glucose Administration in Participants with Diabetes Mellitus

Twelve studies included participants with insulin-dependent diabetes consisting of 19 individual treatment arms. Intravenous glucose was administered in 7 studies with most receiving 0.5-1 g/kg over 5 minutes. Oral glucose (50-100g) was administered in 5 studies. Regular insulin therapy was withheld the morning of glucose administration in all but 2 studies (including 1 case report). An increase in potassium from baseline was seen in 12 treatment arms (63.2%), which ranged from ∼0.2 mmol/L to 1.9 mmol/L.^17^^,^^18^ Six studies included participants with noninsulin diabetes and potassium concentrations increased in 4 of 12 treatment arms (33.3%) with a maximal observed increase of 0.7 mmol/L. Increases in potassium were observed in both oral and intravenous studies. Potassium responses varied widely, with increases more common in insulin-dependent participants.

### Potassium Responses to Glucose Administration in Participants without Diabetes Mellitus

Participants without diabetes were included in 24 studies comprising 47 treatment arms. Intravenous glucose was administered in 7 studies, 5 via bolus administration (0.5-1 g/kg) and 2 via continuous infusion. Oral glucose was administered in 17 studies, most commonly 100 g (range 37.5-150 g).

Statistically significant reductions in serum or plasma potassium were observed in 15 treatment arms following administration of intravenous/oral glucose. An additional 21 treatment arms reported reductions in potassium levels, although statistical significance was not assessed or reported. Five treatment arms showed reductions that were explicitly reported as not statistically significant. The hypokalemic effect ranged from ∼0.15 mmol/L to 0.91 mmol/L.

Six treatment arms reported increases in potassium following glucose administration, but only 2 were reported to be statistically significant.^19^^,^^20^

A summary of the results from each trial arm, stratified by diabetes status, is presented in Table 2.

**Table 2:** Summary of Individual Trial Arm Results, Stratified by Diabetes Status

Reference	Type	(n)	Baseline K+ >5.5 mmol/L	Glucose Dose	Glucose Route	Diabetes Status	RRT	Max Δ K+ (mmol/L)	Statistical Significance	Max Insulin Levels (mU/L)	ECG Data
Ammon et al^17^ (1978)	QES	1	Yes	0.5 g/kg	IV	IDDM	No	1.90	Not reported	NA	No
Ferriss et al^21^ (1979)	QES	5	No	75 g	Oral	IDDM	No	0.18	Not reported	NA	No
Goldfarb et al^22^ (1975)	QES	1	No	51 g	IVI	IDDM	No	1.20	Not reported	NA	No
Goldfarb et al^23^ (1976)	QES	2	No	0.5-1 g/kg	IV	IDDM	No	−0.13	Not reported	NA	No
Goldfarb et al^23^ (1976)	QES	2	No	0.5-1 g/kg	IV	IDDM	No	0.81	Not reported	NA	No
Nicolis et al^24^ (1981)	QES	9	No	100 g	Oral	IDDM	No	1.30	*P* < 0.01	NA	No
Offman et al^25^ (2017)	CS	1	Yes	50 g	IV	IDDM	No	0.90	Not reported	NA	Yes
Perez et al^26^ (1977)	QES	7	Yes	100 g	Oral	IDDM	No	−0.25	Not reported	NA	No
Perez et al^26^ (1977)	QES	6	No	100 g	Oral	IDDM	No	−0.02	Not reported	NA	No
Rado^27^ (1981)	QES	1	Yes	0.5 g/kg	IV	IDDM	No	−0.61	Not reported	NA	No
Rado^27^ (1981)	QES	1	Yes	0.5 g/kg	IV	IDDM	No	0.58	Not reported	NA	No
Rado^27^ (1981)	QES	1	Yes	0.5 g/kg	IV	IDDM	No	−0.60	Not reported	NA	No
Rado^28^ (1981)	QES	4	No	0.5 g/kg	IV	IDDM	No	−0.25	Not reported	93	No
Rado^28^ (1981)	QES	4	No	0.5 g/kg	IV	IDDM	No	0.69	Not reported	89	No
Rado^18^ (1982)	QES	1	No	0.5 g/kg	IV	IDDM	No	0.80	Not reported	NA	No
Rado^18^ (1982)	QES	1	No	0.5 g/kg	IV	IDDM	No	0.20	Not reported	NA	No
Sunderlin et al^29^ (1981)	QES	7	No	100 g	Oral	IDDM	No	−0.16	Not reported	NA	No
Sunderlin et al^29^ (1981)	QES	7	No	100 g	Oral	IDDM	No	0.29	Not reported	NA	No
Viberti^30^ (1978)	QES	8	No	50 g	Oral	IDDM	No	0.47	*P* < 0.005	NA	No
Bae & Kim^31^ (1992)	QES	6	No	100 g	Oral	NIDDM	No	0.44	Not reported	NA	No
Bae & Kim^31^ (1992)	QES	4	No	100 g	Oral	NIDDM	No	−0.34	Not reported	NA	No
Dear et al^32^ (1969)	QES	18	No	1.75 g/kg	Oral	NIDDM	NA	−0.21	Not reported	NA	Yes
Ferriss et al^21^ (1979)	QES	5	No	75 g	Oral	NIDDM	No	−0.39	Not reported	NA	No
Ferriss et al^21^ (1979)	QES	5	No	75 g	Oral	NIDDM	No	−0.25	Not reported	NA	No
Lowenthal et al^33^ (1980)	QES	10	No	NA	Oral	NIDDM	No	−0.32	Not reported	82.6	No
Lowenthal et al^33^ (1980)	QES	10	No	NA	Oral	NIDDM	No	−0.40	Not reported	65.5	No
Rado et al^34^ (1984)	QES	12	No	100 g	Oral	NIDDM	No	−0.20	Not reported	70	No
Rado et al^34^ (1984)	QES	12	No	100 g	Oral	NIDDM	No	−0.22	Not reported	50	No
Rado et al^34^ (1984)	QES	4	No	100 g	Oral	NIDDM	No	0.62	Not reported	50	No
Rado et al^34^ (1984)	QES	4	No	100 g	Oral	NIDDM	No	0.25	Not reported	40	No
Rosenstock et al^35^ (1982)	QES	14	No	50 g	Oral	NIDDM	No	0.70	*P* < 0.01	34	No
Allon et al^36^ (1993)	QES	8	No	50 g	Oral	No diabetes	No	−0.22^a^	*P* < 0.01	100	No
Allon et al^36^ (1993)	QES	8	No	50 g	Oral	No diabetes	No	−0.39^a^	*P* < 0.01	85	No
Allon et al^36^ (1993)	QES	8	No	50 g	Oral	No diabetes	Yes	−0.40^a^	P < 0.01	70	No
Allon et al^36^ (1993)	QES	8	No	50 g	Oral	No diabetes	Yes	−0.57^a^	*P* < 0.01	55	No
Bae & Kim^31^ (1992)	QES	4	No	100 g	Oral	No diabetes	No	−0.36^a^	Not reported	NA	No
Chothia et al^9^ (2014)	RCT	10	Yes	50 g	IV	No diabetes	Yes	−0.50	*P* < 0.001	183	Yes
Dear et al^32^ (1969)	QES	22	No	1.75 g/kg	Oral	No diabetes	NA	−0.25	Not reported	NA	No
Dear et al^32^ (1969)	QES	10	No	1.75 g/kg	Oral	No diabetes	NA	−0.39	Not reported	NA	Yes
Defronzo et al^37^ (1980)	QES	9	No	∼0.88 g/kg	IVI	No diabetes	No	−0.81	Not reported	63^c^	No
Ferriss et al^21^ (1979)	QES	5	No	75 g	Oral	No diabetes	No	−0.24	Not reported	NA	No
Goldfarb et al^23^ (1976)	QES	2	No	0.5-1 g/kg	IV	No diabetes	No	−0.30	Not reported	105	No
Goldfarb et al^23^ (1976)	QES	1	No	0.5-1 g/kg	IV	No diabetes	No	−0.55	Not reported	45	No
Muto et al^38^ (2005)	QES	5	No	150 g	Oral	No diabetes	No	−0.18	NS	73	No
Muto et al^38^ (2005)	QES	13	No	150 g	Oral	No diabetes	Yes	−0.57	*P* < 0.001	72.4	No
Muto et al^38^ (2005)	QES	7	No	75 g	Oral	No diabetes	No	0.18	NS	46.6	No
Muto et al^38^ (2005)	QES	12	No	75 g	Oral	No diabetes	Yes	−0.39	*P* < 0.001	44.5	No
Muto et al^38^ (2005)	QES	13	No	37.5 g	Oral	No diabetes	Yes	−0.30	*P* < 0.001	33	No
Natali et al^39^ (1993)	QES	8	No	75 g	Oral	No diabetes	No	−0.91	Not reported	61	No
Natali et al^39^ (1993)	QES	8	No	75 g	Oral	No diabetes	No	−0.41	Not reported	39	No
Nicolis et al^24^ (1981)	QES	10	No	100 g	Oral	No diabetes	No	−0.80	*P* < 0.01	NA	No
Perez et al^26^ (1977)	QES	6	No	100 g	Oral	No diabetes	No	−0.32	Not reported	NA	No
Rado^20^ (1977)	QES	3	No	1 g/kg	IV	No diabetes	NA	0.00	Not reported	NA	No
Rado^20^ (1977)	QES	3	Yes	1 g/kg	IV	No diabetes	NA	0.65	*P* < 0.05	44	No
Rado^20^ (1977)	QES	6	No	1 g/kg	IV	No diabetes	NA	−0.44	*P* < 0.05	36	No
Rado^19^ (1979)	QES	6	No	1 g/kg	IV	No diabetes	NA	0.20	NS	104.5	No
Rado^19^ (1979)	QES	6	Yes	1 g/ kg	IV	No diabetes	NA	0.46	*P* < 0.01	48.8	No
Rado^19^ (1979)	QES	10	No	1 g/kg	IV	No diabetes	NA	−0.41	*P* < 0.05	44	No
Rado^19^ (1979)	QES	3	No	1 g/kg	IV	No diabetes	NA	0.15	NS	30	No
Rado^40^ (1981)	QES	12	No	100 g	Oral	No diabetes	No	−0.91	*P* < 0.001	NA	No
Rado^40^ (1981)	QES	12	No	100 g	Oral	No diabetes	No	−0.57	*P* < 0.001	NA	No
Rado^28^ (1981)	QES	3	No	1 g/kg	IV	No diabetes	No	−0.39	Not reported	180	No
Rado^28^ (1981)	QES	3	No	1 g/kg	IV	No diabetes	No	−0.36	Not reported	140	No
Rado et al^34^ (1984)	QES	20	No	100 g	Oral	No diabetes	No	−0.37	Not reported	70	No
Rado et al^34^ (1984)	QES	20	No	100 g	Oral	No diabetes	No	−0.76	Not reported	80	No
Rado et al^41^ (1984)	QES	13	No	100 g	Oral	No diabetes	No	−0.62	Not reported	87	No
Rado et al^41^ (1984)	QES	13	No	100 g	Oral	No diabetes	No	−0.28	Not reported	74	No
Rado et al^42^ (1986)	QES	16	No	100 g	Oral	No diabetes	No	−0.40	Not reported	60	No
Rado et al^42^ (1986)	QES	16	No	100 g	Oral	No diabetes	No	−0.20	Not reported	60	No
Rado et al^42^ (1986)	QES	16	No	100 g	Oral	No diabetes	No	−0.30	Not reported	60	No
Rado et al^42^ (1986)	QES	16	No	100 g	Oral	No diabetes	No	0.20	Not reported	60	No
Reynolds et al^43^ (1994)	RCT	13	No	100 g	Oral	No diabetes	No	−0.29^b^	NS	NA	Yes
Rosenstock et al^35^ (1982)	QES	14	No	50 g	Oral	No diabetes	No	−0.15	NS	43	No
Sterns et al^44^ (1981)	QES	6	No	∼25 g	IVI	No diabetes	No	−0.08^a^	NS	17.8^c^	No
Steward et al^45^ (2021)	RCT	8	No	75 g	Oral	No diabetes	No	−0.52^b^	*P* < 0.05	38	No
Sunderlin et al^29^ (1981)	QES	7	No	100 g	Oral	No diabetes	No	−0.41	Not reported	NA	No
Sunderlin et al^29^ (1981)	QES	4	No	100 g	Oral	No diabetes	No	−0.23	Not reported	NA	No
Viberti^30^ (1978)	QES	8	No	50 g	Oral	No diabetes	No	−0.19	*P* < 0.005	NA	No

Abbreviations: IDDM, insulin-dependent diabetes mellitus; IV, intravenous; NIDDM, noninsulin-dependent diabetes mellitus; QES, quasi-experimental study.

a Glucose co-administered with potassium; ΔK+ reported as average change in potassium compared to respective control not receiving glucose.

b Results likely affected by exercise related potassium fluctuations.

c Mean insulin level reported instead of maximum insulin.

### Insulin Levels

A total of 16 studies measured insulin levels following glucose administration (9 oral & 7 intravenous). Peak insulin levels in oral studies ranged from ∼33 mU/L-100 mU/L with glucose doses ranging from 37.5 g to 150 g. Peak insulin levels in intravenous studies were 30 mU/kg-183 mU/kg with most studies using 0.5 g/kg-1 g/kg of glucose.

## Discussion

### Does the Administration of Oral or Intravenous Glucose, in the Absence of Insulin, Lower Serum/Plasma Potassium in Adults?

#### People With Diabetes Mellitus

The findings of this review indicate that the administration of oral or intravenous glucose in patients with diabetes does not reliably lower potassium concentrations and may lead to increases in potassium levels in a substantial proportion of cases.

The administration of hypertonic glucose and or the resultant hyperglycemia is thought to cause an extracellular shift in potassium-rich intracellular water, driven by acute changes in blood osmolarity.^5^ The heterogeneity in potassium response seen in participants with diabetes included in this review may be explained by variations in insulin deficiency, insulin resistance, and multifactorial hyporeninemic hypoaldosteronism. Patients with diabetes are at an increased risk of hyporeninemic hypoaldosteronism, which can result from the use of renin-angiotensin-inhibiting drugs, juxtaglomerular injury caused by diabetic nephropathy, and autonomic neuropathy that impairs renin activation, aldosterone release, and β-adrenergic-mediated potassium influx.^46^^,^^47^

**Figure 3:**
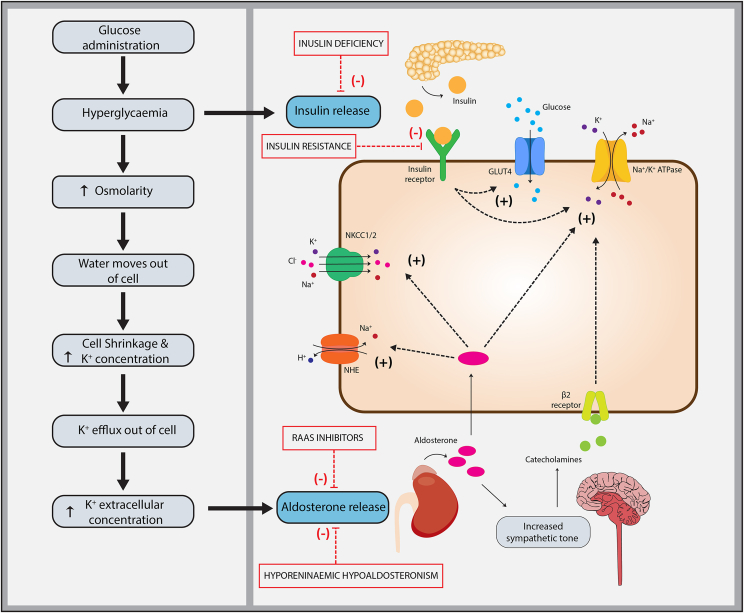
Proposed mechanisms contributing to glucose-induced hyperkalemia in patients with diabetes. Following glucose administration, insulin resistance leads to persistent hyperglycaemia and hyperosmolarity, promoting potassium efflux via osmotic gradients and cell shrinkage. Relative or absolute insulin deficiency limits insulin-mediated stimulation of Na^+^-K^+^-ATPase, impairing cellular potassium uptake. Hyporeninaemic hypoaldosteronism—commonly associated with diabetic nephropathy and autonomic dysfunction—further disrupts potassium homeostasis by reducing aldosterone-mediated regulation of renal excretion and cellular uptake. Aldosterone not only promotes potassium secretion in the distal nephron but also enhances cellular uptake by upregulating Na^+^-K^+^-ATPase and sodium transporters (eg, NHE, NKCC1/2) across various tissues, such as the salivary and sweat glands, colon, airway epithelium, cardiac myocytes, and possibly skeletal muscle. It also acts centrally to increase sympathetic tone, enhancing β_2_-adrenergic receptor–mediated potassium uptake. The use of renin–angiotensin–aldosterone system (RAAS) inhibitors—including ACE inhibitors, angiotensin II receptor blockers, and aldosterone antagonists—further suppresses aldosterone activity, impairing potassium regulation. GLUT4, glucose transporter type 4; NHE, sodium–hydrogen exchanger; NKCC1/2, sodium–potassium–chloride cotransporter isoforms 1 and 2; Na^+^-K^+^-ATPase, sodium–potassium adenosine triphosphatase; RAAS, renin–angiotensin–aldosterone system.

It appears that in some patients with diabetes, these pathophysiological changes lead to an inability to prevent hyperkalemia during physiological stress, such as hyperglycemia or the administration of hypertonic fluids. Patients with type 1 diabetes and insulin-dependent type 2 diabetes are at greatest risk, as both insulin and aldosterone homeostatic mechanisms may be compromised.^23^^,^^24^^,^^30^^,^^31^^,^^35^ Fig 3 summarizes the proposed mechanisms by which patients with diabetes may exhibit a rise—or no significant reduction—in serum potassium following glucose administration. For readers seeking a more detailed overview of potassium homeostasis and the extrarenal effects of aldosterone, we refer to the excellent work by Palmer et al.^3^^,^^4^

A case report captured in this review highlights the potential risk of hypertonic glucose in these at-risk populations.^25^ Cardiac arrest from hyperkalemia was attributed to intravenous glucose administration for hypoglycemia in a patient with insulin-dependent diabetes, stage 3 chronic kidney disease, baseline hyperkalemia, and concurrent spironolactone therapy.

#### People Without Diabetes Mellitus

The evidence supporting glucose-only therapy as a potassium-lowering strategy was stronger in patients without diabetes, with most studies demonstrating reductions in potassium levels ranging from 0.15 mmol/L to 0.91 mmol/L.

The randomized controlled cross-over trial by Chothia et al provides the most robust evidence for glucose-only therapy.^9^ The study compared glucose-only therapy (100 mL of 50% glucose) with IDT (10 units of insulin plus 100 mL of 50% glucose) in 10 patients treated with hemodialysis with hyperkalemia. At 60 minutes, the average potassium reduction was lower in the glucose-only arm (0.5 mmol/L) compared to the IDT arm (0.83 mmol/L). However, this was offset by a markedly improved safety profile: no hypoglycemia occurred in the glucose-only group, whereas 20% of participants in the IDT group experienced hypoglycemia. Glucose levels dropped as low as 2.4 mmol/L and 1.4 mmol/L in specific participants who received IDT, despite the use of a higher-than-usual glucose dose.

Additional support is provided by a hyperinsulinemic euglycemic clamp study by DeFronzo et al,^37^ which demonstrated a 0.81 mmol/L reduction in plasma potassium from baseline in healthy participants over 2 hours. This study is explored in greater detail later in the review.

Oral glucose studies have also shown promise, particularly those by Nicolis et al^24^ and Natali et al,^39^ which reported meaningful reductions in serum potassium among nondiabetic participants. However, many studies did not report statistical significance, and the observed potassium-lowering effects varied widely.

Although most studies demonstrate potassium lowering, some have reported unchanged or elevated levels under pathophysiological conditions. Two studies by the same lead author in the 1970s reported statistically significant increases in potassium following glucose administration.^19^^,^^20^ These studies administered intravenous glucose (50%) 1 g/kg and involved small numbers of patients (n = 6 and n = 3) with baseline hyperkalemia, known renal disease and evidence of a suppressed renin-angiotensin axis. The maximal increase in potassium from baseline was 0.46 mmol/L and 0.65 mmol/L, respectively. The increase in potassium observed in both studies was ameliorated under conditions of low sodium intake that coincided with a dramatic increase in plasma renin and plasma aldosterone levels. This suggests that the risk of a sustained increase in potassium following hypertonic glucose may be related to mineralocorticoid deficiency inhibiting extrarenal potassium handling. Other studies, however, have not demonstrated this response in patients without diabetes who have hypoaldosteronism.^29^

A recent study by Muto et al^38^ found no reduction in serum potassium in healthy participants following oral glucose administration. Glucose doses of 75 g and 150 g were given to 7 healthy individuals and 13 patients treated with hemodialysis, with a 37.5 g dose tested in dialysis patients only. In the hemodialysis group, a dose-dependent potassium reduction was observed (−0.57 mmol/L after 150 g), closely correlated with rising insulin levels. However, healthy participants showed no significant change, despite comparable insulin responses. This result was not explained by baseline potassium levels or changes in aldosterone or catecholamine concentrations, which remained stable.

In contrast, an insulin clamp study by Alvestrand found that insulin-mediated potassium uptake was comparable in participants with renal failure and healthy controls.^48^ Muto et al^38^ attributes this discrepancy to differences between endogenous and exogenous insulin. They proposed that while endogenous insulin can promote an intracellular potassium shift, it may also inhibit renal potassium excretion, offsetting any net reduction in serum potassium in individuals with preserved kidney function. However, this hypothesis is not supported by experimental data, as clamp studies using exogenous insulin have shown reductions in urinary potassium excretion.^49^

A more plausible explanation is that the observed differences reflect the relative magnitude and duration of insulin exposure. In the study by Muto et al,^38^ insulin levels peaked at ∼45 μU/mL before declining, whereas other studies using continuous infusion-maintained levels above 100 μU/mL.^37^^,^^48^ In healthy participants, the transient insulin response in Muto et al^38^ was likely insufficient to induce a measurable potassium shift, while in dialysis patients—who lack urinary potassium losses—a reduction in serum potassium was observed despite similar insulin levels.

Nevertheless, several other oral glucose studies in individuals with preserved renal function have demonstrated reductions in serum potassium.^24^^,^^29^^,^^30^^,^^37^^,^^39^^,^^40^ Further research is needed to confirm these findings and to define the patient characteristics and treatment parameters that influence the efficacy of glucose-only therapy.

### Does Oral or Intravenous Glucose Administration Reliably and Sufficiently Promote Endogenous Insulin Production?

The randomized trial by Chothia et al. found lower serum insulin concentrations in the glucose-only group compared to the IDT arm at all time points. Peak insulin levels measured at 10 minutes were 547.5mU/L in the IDT arm vs 183.7mU/L in the glucose-only arm. Two other studies in hemodialysis patients receiving IDT (not included in this review because of the use of insulin) reported peak insulin levels at 15 minutes of 319 mU/L and 267 mU/L.^50^^,^^51^

Endogenous insulin production following glucose administration is dose dependent and the hypokalemic effect is dependent on the insulin concentrations achieved.^36^^,^^38^^,^^44^ There does however appear to be a ceiling to the hypokalemic effect. The DeFronzo clamp study, previously discussed, involved 17 healthy male participants who received graded infusions of porcine insulin alongside a 20% glucose infusion titrated to maintain euglycaemia.^37^ Insulin infusion rates of 0.25, 0.5, 1, 5, 10 mU/kg/min were chosen to approximate plasma insulin concentrations of 25, 50, 100, 500 and 1000 mU/L respectively. The results demonstrated a significant correlation between plasma potassium concentrations and insulin levels but found the decline in plasma potassium concentration began to plateau at approximately 200mU/L. The decline in plasma potassium concentration at the two lowest insulin doses was similar, at 0.58 mmol/L and 0.62 mmol/L respectively, and similar between the two highest insulin doses, 1.44 mmol/L and 1.54 mmol/L respectively.

In the same study, the potassium-lowering effect of endogenous hyperinsulinemia was examined in 9 participants using a continuous intravenous glucose infusion (without insulin) to elevate and maintain blood glucose at approximately 7 mmol/L above baseline. After 120 minutes, plasma potassium levels had decreased by 0.81 mmol/L. This reduction was statistically greater than those observed with low-dose insulin infusions but smaller than those achieved with high-dose insulin infusions. The average plasma insulin concentration during the glucose infusion was 63 mU/L.

The studies in this review that measured insulin levels after glucose administration consistently reported lower insulin levels compared to studies examining IDT. Notably, nearly all studies used bolus glucose administration, suggesting an opportunity for future research to explore whether a glucose bolus followed by continuous infusion could sustain higher insulin levels over time and enhance potassium-lowering efficacy.

### Does Intravenous Glucose Administration Increase Blood Osmolarity?

Only 2 studies directly measured changes in serum or plasma osmolarity following glucose administration. A 100 g oral glucose study included 10 participants with noninsulin-dependent diabetes and 4 control participants without diabetes.^31^ Among the control group and 4 participants with diabetes, serum potassium decreased similarly, with an average of ∼0.35 mmol/L. However, the remaining 6 participants with diabetes exhibited glucose-induced hyperkalemia, with an average increase of ∼0.44 mmol/L (range 0.1-1.1mmol/L). A strong correlation was observed between increases in serum glucose and serum osmolarity, as well as between serum osmolarity and serum potassium.

Another oral study administered 50 g glucose to 8 participants with insulin-dependent diabetes and 8 control participants without diabetes.^30^ A small but statistically significant increase in potassium occurred in participants with diabetes compared with a small decrease in patients without diabetes. Plasma osmolarity and blood glucose were significantly correlated in the diabetes group, as were blood glucose and plasma potassium. The maximum change in plasma osmolarity was +5.14 mOsm/kg in participants with diabetes vs +1.7 mOsm/kg in participants without diabetes. Although the change in osmolarity in participants without diabetes was statistically significant at 30 minutes, it was not associated with a rise in plasma potassium.

Endogenous insulin production appears to protect against the rise in potassium following hypertonic glucose administration. This is further supported by studies demonstrating significantly increased plasma potassium concentrations in patients without diabetes following hypertonic saline (2.5% and 5%) administration—a solution that does not stimulate endogenous insulin production.^52^

Another study measured C-peptide concentrations following IDT administration to patients treated with hemodialysis as a marker of endogenous insulin production.^50^ The authors speculated that endogenous insulin production would overwhelm any short-lived extracellular potassium shift caused by the hypertonicity of the glucose solution. Average plasma potassium fell by 0.51 mmol/L at 15 minutes (the earliest time point) and by 0.76 mmol/L at 60 minutes. C-peptide concentrations increased from 3.83 nmol/L at baseline to 5.33 nmol/L at 30 minutes.

Data from the randomized controlled trial by Chothia et al^9^ provides data to support this view, observing that at 10 minutes post-administration, one patient in the glucose-only group exhibited a higher serum potassium level (mean increase 0.2 mmol/L), compared to three patients in the IDT group (mean increase 0.4 mmol/L). However, average potassium levels did not increase at any time point in either group.

These findings suggest that while endogenous insulin production may attenuate the magnitude and duration of potassium increases, it does not entirely prevent small, transient rises in potassium. Importantly, transient increases in potassium can also occur when co-administered with exogenous insulin as seen with standard IDT.

### Research Gaps and Future Direction

This scoping review identified several research gaps and areas for future investigation regarding glucose-only therapy for hyperkalemia management. To date, only one small randomized controlled trial has compared glucose-only therapy to IDT in patients with hyperkalemia. Of the studies reviewed, only 3 included nondiabetic participants with baseline potassium levels above 5.0 mmol/L, and just one study — the aforementioned randomized controlled trial—involved participants with potassium levels exceeding 6.0 mmol/L. As a result, there is limited evidence on the efficacy of glucose-only therapy, particularly regarding its impact on ECG changes.

No studies examined the impact of glucose-only therapy in combination with other potassium-lowering therapies, such as β-agonists or potassium-binding resins. The potassium-lowering effect of salbutamol plus glucose-only therapy is particularly worth exploring, as salbutamol plus IDT has been shown to be superior to either agent alone.^53^ Only 3 studies included patients on renal replacement therapies, a critical demographic given their elevated risk of hyperkalemia, but the limited data appear promising.

No studies evaluated safety endpoints such as thrombophlebitis or extravasation, despite 50% glucose being hypertonic and potentially irritating to vessels.^54^^,^^55^ Importantly, the risk of infusion-related adverse events in future studies of glucose-only therapy would likely be comparable to standard care, as 50% glucose is routinely co-administered with insulin to prevent hypoglycemia during hyperkalemia treatment, although empirical data are lacking.

Given these research gaps, future randomized trials should compare glucose-only therapy plus salbutamol with IDT plus salbutamol in patients with potassium levels > 5.5 mmol/L. Outcomes should include change in serum/plasma potassium, ECG changes, and adverse effects to evaluate efficacy and safety. Future work should also explore where glucose-only therapy might be best utilized. This may include resource limited settings such as prehospital, remote areas or emergencies where insulin is unavailable due refrigeration or supply issues. Glucose-only therapy may also offer a better risk-benefit profile for patients with moderate hyperkalemia who have risk factors for insulin-induced hypoglycemia (eg, lower pretreatment blood glucose, renal impairment, and low body weight).^7^ Oral glucose strategies might be useful in circumstances where intravenous access is impractical or where close blood glucose monitoring for 6 hours is not feasible, but further study is needed.

### Limitations

This scoping review has several limitations. Many included studies were over 30 years old, lacked detailed patient data, and did not report statistical analysis. Additionally, raw data were often unavailable, requiring data extraction from figures, which may introduce error. Given the heterogeneity of interventions, populations, and outcome reporting, findings should be interpreted with caution and cannot be generalized to all patient groups.

## Conclusion

Glucose-only therapy may be a safe, effective option for hyperkalemia in people without diabetes, avoiding the risk of hypoglycemia. However, well-designed clinical trials are needed to confirm its efficacy and define its optimal use. In contrast, glucose-only therapy is not recommended in patients with diabetes, given the inconsistent findings and the potential for glucose-induced hyperkalemia.
