# Supplementary material for: Glucose-only Therapy for Potassium Reduction: A Scoping Review
Source: Kidney Med. 2025 Oct 29;8(1):101161. doi: 10.1016/j.xkme.2025.101161 (PMC12880623; doi:10.1016/j.xkme.2025.101161)
Supplement: Supplementary File (PDF) — Tables S1-S3. [file mmc1.pdf]

Table S1.

SECTION	ITEM	PRISMA-ScR CHECKLIST ITEM	REPORTED ON PAGE #
**TITLE**			
Title	1	Identify the report as a scoping review.	1
**ABSTRACT**			
Structured summary	2	Provide a structured summary that includes (as applicable): background, objectives, eligibility criteria, sources of evidence, charting methods, results, and conclusions that relate to the review questions and objectives.	2
**INTRODUCTION**			
Rationale	3	Describe the rationale for the review in the context of what is already known. Explain why the review questions/objectives lend themselves to a scoping review approach.	4-5
Objectives	4	Provide an explicit statement of the questions and objectives being addressed with reference to their key elements (e.g., population or participants, concepts, and context) or other relevant key elements used to conceptualize the review questions and/or objectives.	5
**METHODS**			
Protocol and registration	5	Indicate whether a review protocol exists; state if and where it can be accessed (e.g., a Web address); and if available, provide registration information, including the registration number.	6
Eligibility criteria	6	Specify characteristics of the sources of evidence used as eligibility criteria (e.g., years considered, language, and publication status), and provide a rationale.	7
Information sources*	7	Describe all information sources in the search (e.g., databases with dates of coverage and contact with authors to identify additional sources), as well as the date the most recent search was executed.	7
Search	8	Present the full electronic search strategy for at least 1 database, including any limits used, such that it could be repeated.	7
Selection of sources of evidence†	9	State the process for selecting sources of evidence (i.e., screening and eligibility) included in the scoping review.	7-8
Data charting process‡	10	Describe the methods of charting data from the included sources of evidence (e.g., calibrated forms or forms that have been tested by the team before their use, and whether data charting was done independently or in duplicate) and any processes for obtaining and confirming data from investigators.	8
Data items	11	List and define all variables for which data were sought and any assumptions and simplifications made.	8
Critical appraisal of individual sources of evidence§	12	If done, provide a rationale for conducting a critical appraisal of included sources of evidence; describe the methods used and how this information was used in any data synthesis (if appropriate).	9, Table S3
Synthesis of results	13	Describe the methods of handling and summarizing the data that were charted.	9
**RESULTS**			
Selection of sources of evidence	14	Give numbers of sources of evidence screened, assessed for eligibility, and included in the review, with reasons for exclusions at each stage, ideally using a flow diagram.	9-10, Figure 1
Characteristics of sources of evidence	15	For each source of evidence, present characteristics for which data were charted and provide the citations.	14-16
Critical appraisal within	16	If done, present data on critical appraisal of included sources of evidence (see item 12).	Table S3

sources of evidence			
Results of individual sources of evidence	17	For each included source of evidence, present the relevant data that were charted that relate to the review questions and objectives.	9-16
Synthesis of results	18	Summarize and/or present the charting results as they relate to the review questions and objectives.	9-12
**DISCUSSION**			
Summary of evidence	19	Summarize the main results (including an overview of concepts, themes, and types of evidence available), link to the review questions and objectives, and consider the relevance to key groups.	17-25
Limitations	20	Discuss the limitations of the scoping review process.	25
Conclusion	21	Provide a general interpretation of the results with respect to the review questions and objectives, as well as potential implications and/or next steps.	26
**FUNDING**			
Funding	22	Describe sources of funding for the included sources of evidence, as well as sources of funding for the scoping review. Describe the role of the funders of the scoping review.	27

Table S1 - Preferred Reporting Items for Systematic reviews and Meta-Analyses extension for Scoping Reviews (PRISMA-ScR) Checklist.

JBI = Joanna Briggs Institute; PRISMA-ScR = Preferred Reporting Items for Systematic reviews and Meta-Analyses extension for Scoping Reviews.
* Where sources of evidence (see second footnote) are compiled from, such as bibliographic databases, social media platforms, and Web sites.
† A more inclusive/heterogeneous term used to account for the different types of evidence or data sources (e.g., quantitative and/or qualitative research, expert opinion, and policy documents) that may be eligible in a scoping review as opposed to only studies. This is not to be confused with information sources (see first footnote).
‡ The frameworks by Arksey and O’Malley (6) and Levac and colleagues (7) and the JBI guidance (4, 5) refer to the process of data extraction in a scoping review as data charting.
§ The process of systematically examining research evidence to assess its validity, results, and relevance before using it to inform a decision. This term is used for items 12 and 19 instead of "risk of bias" (which is more applicable to systematic reviews of interventions) to include and acknowledge the various sources of evidence that may be used in a scoping review (e.g., quantitative and/or qualitative research, expert opinion, and policy document).
From: Tricco AC, Lillie E, Zarin W, O'Brien KK, Colquhoun H, Levac D, et al. PRISMA Extension for Scoping Reviews (PRISMAScR): Checklist and Explanation. Ann Intern Med. 2018;169:467–473. doi: 10.7326/M18-0850.

Database	Search Strategy
PubMed (PubMed)	("Insulin"[Mesh] OR "Glucose"[Mesh] OR "insulin"[tiab] OR "dextrose"[tiab] OR "glucose"[tiab])<br>**AND**<br>("Hyperkalemia"[Mesh] OR "hyperkalaemia"[ti] OR "hyperkalemia"[ti] OR "serum potassium"[ti])
Embase (Elsevier)	('insulin'/exp/mj OR 'glucose'/exp/mj OR "insulin":ti,ab OR "dextrose":ti,ab OR "glucose":ti,ab)<br>**AND**<br>('hyperkalemia'/exp/mj OR "hyperkalaemia":ti OR "hyperkalemia":ti OR "serum potassium":ti)<br>**AND** ([article]/lim OR [article in press]/lim OR [review]/lim)
Web of Science (Clarivate)	(TI=("insulin" OR "dextrose" OR "glucose") OR AB=("insulin" OR "dextrose" OR "glucose"))<br>**AND**<br>(TI=("hyperkalaemia" OR "hyperkalemia" OR "serum potassium"))
Cochrane Library & CENTRAL (Wiley)	MeSH descriptor: [Insulins] explode all trees<br>MeSH descriptor: [Glucose] explode all trees<br>("insulin" OR "dextrose" OR "glucose"):ti<br>("insulin" OR "dextrose" OR "glucose"):ab<br>#1 OR #2 OR #3 OR #4<br>MeSH descriptor: [Hyperkalemia] explode all trees<br>("hyperkalaemia" OR "hyperkalemia" OR "serum potassium"):ti<br>#6 OR #7<br>#5 AND #8

Table S2: Detailed Search Strategies

Study Author, Year	Study Title	Was the timing of the intervention and outcome measurement clearly reported and appropriate for observing an effect?	Did the study include any form of control intervention (e.g., IDT or placebo?)	Were participant groups sufficiently described?	Were the participants included in any comparisons receiving similar treatment/care, other than the exposure or intervention of interest?	Were outcomes (e.g., serum potassium) measured using consistent, valid, and reliable methods?	Were outcomes reported with sufficient detail for interpretation (e.g., numerical values, statistical significance, measures of variation)?
Allon et al, 1993	Glucose Modulation of the Disposal of an Acute Potassium Load in Patients With End-Stage Renal Disease	Yes	No	Yes	No	Yes	Yes
Ammon et al, 1978	Glucose-Induced Hyperkalemia with Normal Aldosterone Levels	Yes	No	Yes	NA	Unclear	No
Bae HY, Kim HJ, 1992	Heterogeneous changes of serum pottasium levels in NIDDM patients on oral glucose load	Yes	No	Yes	Yes	Yes	No

Chothia et al, 2014	Bolus adminsitration of intravenous glucose in the treatment of hyperkalaemia: A Randomised Controll Trial	Yes	Yes	Yes	Yes	Yes	Yes
Dear et al, 1969	Changes in electrocardiogram and serum potassium values following glucose ingestion	Yes	No	Yes	Unclear	Yes	No
Defronzo et al, 1980	Effect of graded doses of insulin on splanchnic and peripheral potassium metabolism in man	Yes	No	Yes	Yes	Yes	No
Ferriss et al, 1979	Hypertension, hyperkalaemia and abnormalities of the renin-angiotensin system in diabetes mellitus	No	No	Yes	Unclear	Unclear	No
Goldfarb et al, 1976	Acute Hyperkalaemia Induced by Hyperglycemia: Hormonal Mechanisms	Yes	No	Yes	No	Unclear	No
Goldfarb et al, 1975	Paradoxical glucose-induced hyperklaemia. Combined aldosterone-insulin deficiency.	Yes	Yes	No	NA	Unclear	No

Lowenthal et al, 1980	Effects of amiloride on oral glucose loading, serum potassium, renin and aldosterone in diet-controlled diabetes	No	No	No	Unclear	Unclear	No
Muto et al, 2005	Effect of oral glucose adminsitration on serum potassium concentration in haemodialysis patients	Yes	No	No	No	Yes	Yes
Natali et al, 1993	Relationship between insulin release, antinatiruesis and hypokalaemia after glucose ingestion in normal and hypertensive man	No	No	Yes	Yes	Yes	No
Nicolis et al, 1981	Glucose-Induced hyperkalaemia in diabetic subjects	Yes	No	Yes	Yes	Yes	No
Offman et al, 2017	Hyperkalaemia and cardiac arrest associated with glucose replacement in a patient on spironolactone	NA	NA	Yes	NA	Unclear	NA
Perez et al, 1977	Potassium homeostasis in chronic diabetes mellitus	Yes	No	No	Yes	Yes	No
Rado, 1977	Effects of mineralcorticoids on the paradoxical glucose-induced hyperkalaemia in nondiabetic patients	Yes	No	No	Unclear	Unclear	No

	with selective hypoaldosteronism						
Rado et al, 1979	Glucose-induced paradoxical hyperkalemia in patients with suppresion of the renin-aldosterone system: prevention by sodium depletion	Yes	No	No	No	Yes	Yes
Rado, 1981	Influence of posture on serum potassium changes during standard glucose tolerance test	Yes	No	No	Yes	Unclear	No
Rado, 1981	Reversal of glucose-induced hyperkalaemia by sodium restriction in normaldosteronemic diabetes mellitus	Yes	No	No	No	Unclear	No
Rado et al, 1981	Glucose-induced hyperkalaemia in normaldosteronemic diabetes associated with hyperthyroidism	No	No	Yes	NA	Unclear	No
Rado, 1982	A possible role for human growth hormone (HGH) in the protection from glucose-induced hyperkalaemia in	Yes	No	Yes	NA	Unclear	No

	normaldosteronemic diabetes						
Rado et al, 1984	Effect of Body Posture on the Serum Potassium Response to Glucose in Healthy Subjects, Diabetics and Hyperthyroid Patients'	Yes	No	No	No	Yes	No
Rado et al, 1984	Effect of posture and a mineralocorticoid on the glucose-induced hypokalemia in man	Yes	No	Yes	Yes	Yes	Yes
Rado et al, 1986	Interplay of various factors in glucose-induced hyperkalaemia during captopril treatment	Yes	No	No	Yes	Yes	No
Reynolds et al, 1994	Influence of postexercise glucose ingestion upon serum potassium levels and ECG function	Yes	No	Yes	Yes	Yes	No
Rosenstock et al, 1982	Effect of Acute Hyperglycaemia on Plasma Potassium and Aldosterone Levels in Type 2 (Non-Insulin-Dependent) Diabetes	Yes	No	Yes	Unclear	Yes	No

Sterns et al, 1981	The disposition of intravenous potassium in normal man: the role of insulin	Yes	No	No	Yes	Yes	Yes
Steward et al, 2021	A single oral glucose load decreases arterial plasma [K+] during exercise and recovery	Yes	Yes	No	Yes	Yes	No
Sunderlin et al, 1981	The renin-angiotensin-aldosterone system in diabetic patients with hyperkalaemia	Yes	No	No	Yes	Yes	No
Viberti, 1978	Glucose-induced hyperkalaemia - hazard for diabetics	Yes	No	No	Yes	Yes	No

Table S3: Risk of Bias Assessment for Included Studies

Adapted from: Barker TH, Habibi N, Aromataris E, Stone JC, Leonardi-Bee J, Sears K, et al. The revised JBI critical appraisal tool for the assessment of risk of bias quasi-experimental studies. JBI Evid Synth. 2024;22(3):378-88.
